# Letter from the Editor-in-Chief

**DOI:** 10.1289/ehp.1408430

**Published:** 2014-04-01

**Authors:** Hugh A. Tilson

**Affiliations:** Editor-in-Chief, *Environmental Health Perspectives*, E-mail: tilsonha@niehs.nih.gov

The first day of August 2014 will be a big day for me. I have decided to retire after nearly 40 years of federal service, and *Environmental Health Perspectives* (*EHP)* will need a new editor-in-chief. It has been a real privilege to lead this important scientific journal since 2008. I believe in *EHP*, the science it supports, and the communities it serves, and I am proud to have had a part in the journal’s continued success and growing impact.

In my inaugural editorial as editor-in-chief (Tilson 2008), I wrote that *EHP* must adhere to the principles of independence, transparency, and balance and be prepared to recognize emerging themes in order to attract a wider audience and have an impact on the field. Adherence to these standards has served the journal well. In 6 years, the journal’s impact factor increased from 5.86 to 7.26. *EHP* is one of the top-ranked journals in Public, Environmental, and Occupational Health and in Environmental Sciences. The journal receives nearly 1,500 research manuscripts each year and has an acceptance rate of 15%. We have more than 40 internationally recognized members of the Board of Associate Editors and more than 100 active members of the Editorial Review Board. The journal routinely publishes highly recognized news articles on a variety of emerging topics in environmental health research. In collaboration with the Shanghai Center for Disease Control and Prevention, we also publish news and other articles in a bimonthly issue of *EHP Chinese Edition*. The journal established and cultivated a thriving social media presence on Facebook and Twitter, and updated its format to provide easy access on personal communication devices. In 2013, *EHP* was one of the first environmental health journals to publish exclusively online, with the entire journal available electronically and free of charge.

I thank the leadership of the National Institute of Environmental Health Sciences (NIEHS) for providing continuous and enthusiastic support and for making good on its commitment to editorial independence and unfettered scientific peer review. The *EHP* staff members also deserve my gratitude and thanks. They are of some of the most dedicated and passionate individuals I have ever known. I believe that the articles and news we published have made a significant contribution to improving human health and have provided the best available information for decision makers and policy makers worldwide. I am confident that my successor will find the NIEHS and the *EHP* staff eager to carry on this tradition of excellence.

The NIEHS is organizing a search committee to recruit my replacement. I have agreed to assist with the transition and, if necessary, will happily work as a volunteer until a new editor-in-chief is named. In the meantime, *EHP* will continue to receive manuscripts and publish research and news stories each month. Keep sending us your science, and by all means, keep reading *EHP* !
